# The association between all-cause mortality with drinking habits and water sources: a nationwide longitudinal study on Chinese elderly

**DOI:** 10.7189/jogh.15.04120

**Published:** 2025-09-12

**Authors:** Shisi Shen, Ning Ma, Tingting Wu, Yang Xiong, Jialu Yang, Xiaoai Wu, Xianhong Xiang

**Affiliations:** 1 Departments of Obstetrics and Gynecology, Peking Union Medical College Hospital, Chinese Academy of Medical Sciences and Peking Union Medical College, Beijing, China; 2 School of Public Health, Capital Medical University, Beijing, China; 3 School of Public Health and Management, Chongqing Medical University, Chongqing, China; 4 The West China Hospital, Sichuan University, Chengdu, China; 5 Division of Prevention and Community Health, National Center for Cardiovascular Disease, National Clinical Research Center of Cardiovascular Disease, State Key Laboratory of Cardiovascular Disease, Fuwai Hospital, Peking Union Medical College & Chinese Academy of Medical Sciences, Beijing, China; 6 Department of Nuclear Medicine, Laboratory of Clinical Nuclear Medicine, West China Hospital, Sichuan University, Chengdu, China; 7 Department of Interventional Radiology, The First Affiliated Hospital of Sun Yat-sen University, Guangzhou, China

## Abstract

**Background:**

Few studies have reported on the association between drinking habits, water sources and all-cause mortality among the elderly, who are susceptible to toxic environmental factors. We aimed to address this gap by conducting a longitudinal study among the Chinese population.

**Methods:**

We conducted a 16-year longitudinal study using data of individuals aged >65 years at baseline enrolled in the Chinese Longitudinal Healthy Longevity Study. A formal questionnaire was used to collect data on drinking habits and water sources. The former to whether participants consumed boiled or unboiled water, while the latter queried the use of well water, surface water, spring water, and tap water. We used Cox proportional hazard adjusted for sociodemographic factors, lifestyle, and common diseases to calculate the risk of all-cause mortality associated with drinking water. We further conducted subgroup analyses to evaluate potential interaction effects.

**Results:**

We used data on 15 664 individuals, among whom 4472 men and 6166 women died from any reason. Participants who drank unboiled water were more likely than those who drank boiled water to eventually reach a high risk of all-cause mortality (hazard ratio (HR) = 1.14; 95% confidence interval (CI) = 1.06–1.23). Compared to drinking well water, drinking tap water in childhood (HR = 0.80; 95% CI = 0.68–0.95), being around 60 years of age (HR = 0.81; 95% CI = 0.76–0.86), and at present (HR = 0.90; 95% CI = 0.86–0.95) were all associated with lower risks of all-cause mortality. Drinking surface water in childhood was also related to a lower risk of all-cause mortality (HR = 0.94; 95% CI = 0.90–0.98). However, drinking spring water was not associated with all-cause mortality across the entire lifespan in the total sample.

**Conclusions:**

Drinking unboiled water was associated with a higher risk of all-cause mortality. In comparison to well water, tap water emerged as a safer and healthier option for the elderly Chinese population throughout their whole life cycle.

Water, constituting about 60% of the human body, is essential for bodily functions [1]. In China, however, water pollution has become a pressing public issue due to increasing pollutant discharge from industrial, agricultural, and household sources [2–4]. In the 2012 Yale Environmental Performance Index, the country ranked 120 out of 180 countries in terms of its poor water quality [2]. Half of its population lacks access to safe drinking water, with over 500 million rural residents relying on contaminated sources. Industrial waste, chemical fertilisers, and raw sewage contribute to China’s USD 69 billion pollution-related annual losses. These pollutants are associated with significantly elevated cancer risks, showing mortality risk ratios of 1.13 for all-cancer, 1.82 for stomach cancer, 1.15 for lung cancer, 1.74 for colon cancer, 2.23 for rectal cancer, and 2.02 for colorectal cancer compared to unexposed regions in China [4–6].

Long-term consumption of unprocessed water (*e.g.* well, surface, and spring) poses significant health risks. Studies have linked contaminated water to water-borne diseases, such as typhoid fever, diarrhoea, gastroenteritis, and cholera [7–10]. The World Health Organization (WHO) reported that contamination of water and food has resulted in an estimated 2.9 million cholera cases and 95 000 deaths annually worldwide [11]. Inadequate sanitation, unsafe water, and poor hygiene account for about 88% of diarrhoea-associated deaths in developing countries, with 801 000 children dying annually [12,13]. Diarrhoea also negatively impacts growth and cognitive development, reducing survival quality and life expectancy [12]. Furthermore, drinking contaminated water has been associated with increased risks of cancer, including lung, stomach, esophageal, and colorectal cancers [5,6,14].

Beyond the risks associated with unprocessed water, how water is consumed can also significantly impact human health. Improper hydration practices, similar to exposure to polluted water sources, may pose significant physiological risks. In China, boiled water is traditionally advocated for as the optimal hydration choice, supported by both media and nutritional literature [15]. However, the specific health benefits of exclusively consuming boiled water lack empirical substantiation [16]. Many Western countries have been promoting cold water, which temporarily increases metabolism and heat production and even triggers the release of stress hormones, catecholamines, and endorphins, enhancing alertness, mood, and mental health [17].

Previous research has extensively examined the associations between substantial water intake and various diseases. Cold water therapy has demonstrated benefits for cardiovascular and metabolic risk factors, including blood lipid profiles, homocysteine concentrations and insulin sensitivity [17]. Epidemiological studies have, for example, found an unexpected inverse correlation between cardiovascular event mortality and water hardness [18,19]. Considering that boiling water reduces mineral content and hardness, few studies have explored the association between drinking habits, water sources, and all-cause mortality in older people, especially in low- and middle-income countries. Large-scale cohort studies are urgently needed to investigate the impact of drinking habits and water sources on all-cause mortality.

Recent studies have suggested that long-term exposure to ambient chemicals can also lead to diseases later in life, particularly in older populations [20]. As China’s population ages, the mortality rate related to unsafe drinking water among the elderly is likely to increase rapidly [21]. We hypothesised that drinking unboiled water would be associated with an elevated risk of all-cause mortality among the elderly Chinese, while drinking tap water would have a protective influence on all-cause mortality throughout the lifespan, including at childhood, at 60 years, and at present (*i.e.* at the time of data collection). To verify this hypothesis, we investigated it within the framework of a large-scale cohort study, specifically drawing data from the Chinese Longitudinal Healthy Longevity Study (CLHLS).

## METHODS

### Study design and subjects

The CLHLS is a dynamic longitudinal study conducted every two to four years, covering 85% of China’s elderly population across 22 provinces. From 1998 to 2000, it recruited participants aged ≥80 using multistage, stratified cluster sampling. Since 2002, however, participants aged 65–79 have also been included. The study collects data on socioeconomic characteristics, lifestyle, social interactions, residential environment, cognitive function, and physical/mental conditions.

Six survey waves have been conducted, with follow-ups for living participants and interviews with relatives of deceased participants to gather information on the causes of death and health conditions prior to death. Questionnaires include ‘survivors’ (for living participants) and ‘deceased’ (for relatives of deceased participants) sections [22]. We used datasets from the 2002, 2005, 2008, 2011, 2014, and 2018 waves, focussing on participants aged ≥65. Of the 16 064 individuals selected in 2002, 15 664 had complete data on drinking habits, water sources, and covariates. After excluding 188 participants with missing or ambiguous data and 212 who relocated, we established a fixed cohort of 15 664 individuals.

### Death assessment

The CLHLS research team followed interviewees from baseline until death, dropout, or the latest wave of surveys. For example, the CLHLS research team obtained the date of participants' death *via* interviews with family members or local doctors during subsequent surveys in the cohort. As cause-specific mortality data in the CLHLS might be missing and ambiguous, we did not include them in this study [23].

### Assessment of drinking habits and water sources

The CLHLS research team verified participants’ drinking habits by asking them ‘what kind of water do you usually drink?’ (boiled or unboiled), and identified water sources across the participants' life course with three questions: ‘what source of water did you mainly drink from in your childhood?’, ‘what source of water did you mainly drink from around the age of 60?’, and ‘what source of water do you mainly drink from at present?’. The options included well water, surface water (from the rivers, lakes, ponds, and pools), spring water, and tap water.

### Covariates

We adjusted for several potential confounders, such as sociodemographic characteristics, lifestyle factors, and health status. Sociodemographics included age, sex, residential place (rural or urban), education level (illiterate, primary school, or high school and above), and marital status (never married/single or married and living with spouse). Lifestyle factors included smoking status (never, previously, or currently), drinking status (never, previously, or currently), dietary diversity scores (low, medium, or high) and social activity (never, sometimes, or always). Health status included body weight (in kg), common diseases (*i.e.* hypertension, diabetes, heart disease, stroke, pneumonia, and tuberculosis (yes or no), cognitive function (measured by the Mini-Mental State Examination (MMSE)), and depression status (measured by a five-item scale).

### Statistical analysis

We expressed continuous variables as means and standard deviations and categorical variables as numbers and percentages. We used multivariable Cox regression models to examine hazard ratios (HRs) between drinking habits, water sources and all-cause mortality. Boiled water and well water served as references for drinking habits and water sources, respectively. We tested the proportional hazards assumption before carrying out the analysis. We conducted subgroup analyses stratified by sex, residence area, and disease status. For multivariate models, we adjusted for age, sex, weight, education, residence, marital status, alcohol consumption, smoking, physical activity, social activity, food scores, cognitive impairment, depression, and common diseases. We estimated the crude all-cause mortality rate (per 1000 person-years) across various drinking habits and water sources. An HR > 1 indicates increased mortality risk in the experimental group compared to the control, while an HR < 1 suggests reduced risk.

We performed five sensitivity analyses to address the potential influence of dropout. Firstly, we applied multiple imputations with five replications and Monte Carlo simulations to handle missing covariates, using a chained equation approach under a Gaussian normal distribution assumption [24]. We used continuous iterative interpolation to estimate missing values and performed regression analyses on five imputed datasets to ensure robustness. Secondly, we excluded participants who died in 2005 (the second wave) to mitigate bias from undocumented medical conditions. Thirdly, we excluded individuals with common diseases to minimise the potential effects of these diseases on the outcome. Fourthly, we incorporated a time-varying analysis to account for temporal variations in drinking habits. Finally, we further adjusted the models for income levels and geographic regions to address socioeconomic and regional confounding effects.

We used Stata, version 16.1 (StataCorp, College Station, Texas, USA) for the analyses, with statistical significance set at a two-sided *P*-value < 0.05.

## RESULTS

### Characteristics of all participants at baseline

We included 15 663 participants at baseline, with an average age of 86.5 years and weight of 49.0 kg (**Table 1**). Among them, 8985 (57.4%) were female and 6678 (42.6%) were male. Over a median follow-up of six years, 10 638 participants died (4472 males, 6166 females). Regarding drinking habits, 14 952 participants habitually drank boiled water. In childhood, 9361 drank well water, 5209 drank surface water, 703 drank spring water, and 390 drank tap water. At around the age of 60 years, 8200 drank well water, 2480 drank surface water, 613 drank spring water, and 4370 drank tap water. At the time of data collection, 5547 drank well water, 232 drank surface water, 509 drank spring water, and 9375 drank tap water. Most participants were never married or were single (n = 11 075, 70.7%) and did not participate in social activities (n = 13 615, 86.9%). Almost two-thirds were illiterate (n = 9701, 61.9%), had never smoked (n = 10 211, 65.3%), and did not drink alcohol (n = 10 458, 66.9%). Over half of the participants lived in rural areas (n = 8495, 54.2%) and reported low leisure-time physical activity levels (n = 8949, 57.2%), while just under half (n = 6406, 40.9%) had low dietary diversity scores. More than half of the participants had cognitive impairment (n = 7421, 56.6%), while the mean depression score was 11.8. Regarding common diseases, 2368 (15.8%) had hypertension, 360 (2.4%) had diabetes, 1388 (9.3%) experienced a heart attack, 833 (5.5%) had stroke/cardiovascular disease, 2035 (13.5%) had pneumonia, and 119 (0.8%) had tuberculosis. We also performed comparisons of different drinking habits and water sources at specific life cycles, as well as the regional distribution of varying drinking habits and water sources at the age of 60 (Tables S1–6 in the **Online Supplementary Document**).

**Table 1:** Baseline characteristics of participants (n = 15 663)*

Age (in years), x̄ (SD)	86.5 (11.6)
**Weight, x̄ (SD)**	49.0 (10.8)
**Sex**	
Male	6678 (42.6)
Female	8985 (57.4)
**Education level**	
Illiterate	9701 (61.9)
Primary school	4406 (28.1)
High school or above	1556 (9.9)
**Living area**	
Rural	8495 (54.2)
Urban	7168 (45.8)
**Marital status**	
Never married or single	11 075 (70.7)
Married and living with spouse	4588 (29.3)
**Drinking habits**	
Boiled water	14 952 (95.5)
Unboiled water	711 (4.5)
**Water sources at childhood**	
Well	9361 (59.8)
Surface	5209 (33.3)
Spring	703 (4.5)
Tap water	390 (2.5)
**Water sources at 60 years**	
Well	8200 (52.4)
Surface	2480 (15.8)
Spring	613 (3.9)
Tap water	4370 (27.9)
**Water sources at present**	
Well	5547 (35.4)
Surface	232 (1.5)
Spring	509 (3.2)
Tap water	9375 (59.9)
**Drinking status**	
Never	10 458 (66.9)
Previously	1972 (12.6)
Currently	3195 (20.4)
**Smoking status**	
Never	10 211 (65.3)
Previously	2545 (16.3)
Currently	2879 (18.4)
**Physical activity**	
Low	8949 (57.2)
Medium	1787 (11.4)
High	4897 (31.3)
**Social activity**	
Never	13 615 (86.9)
Sometimes	1767 (11.3)
Always	281 (1.8)
**Food scores**	
Low	6406 (40.9)
Medium	4450 (28.4)
High	4807 (30.7)
**Cognitive impairment**	
No	5695 (43.4)
Yes	7421 (56.6)
**Depression, x̄ (SD)**	11.8 (3.3)
**Hypertension**	
No	12 643 (84.2)
Yes	2368 (15.8)
**Diabetes**	
No	14 597 (97.6)
Yes	360 (2.4)
**Heart attack**	
No	13 591 (90.7)
Yes	1388 (9.3)
**Stroke/CVD**	
No	14 208 (94.5)
Yes	833 (5.5)
**Pneumonia**	
No	13 064 (86.5)
Yes	2035 (13.5)
**Tuberculosis**	
No	14 903 (99.2)
Yes	119 (0.8)

CVD – cardiovascular disease, SD – standard deviation, x̄ – mean

*Presented as n (%) unless specified otherwise.

### Associations between drinking habits and the risk of all-cause mortality

Drinking unboiled water presented a 14% higher risk of death than drinking boiled water (**Table 2**), meaning they had a higher risk of all-cause mortality (HR = 1.14; 95% CI = 1.06–1.23). Additionally, Cox models stratified by sex, living area, and disease status produced consistent results with the total sample (*P* < 0.05).

**Table 2:** Cox regression for all-cause mortality with drinking habits*

	Boiled water	Unboiled water, HR (95% CI)
**Number of deaths, n**	10 079	559
**Person-years, n**	133 590	5921
**Incident rate, ‰**	75.45	94.41
**All participants**	ref	1.14 (1.06–1.23)
**Living area**		
Rural	ref	1.19 (1.01–1.42)
Urban	ref	1.13 (1.04–1.23)
**Sex**		
Male	ref	1.16 (1.03–1.30)
Female	ref	1.13 (1.02–1.25)
**Disease**		
No	ref	1.11 (1.01–1.22)
Yes	ref	1.23 (1.06–1.41)

CI – confidence interval, HR – hazard ratio, ref – reference

*Model adjusted for baseline sex. age, residence, smoking, drinking alcohol, dietary diversity score, weight, marital status, social activity and education, depression, MMSE, and disease.

### Association of kinds of water sources at childhood with the risk of mortality

Compared to participants who drank well water in childhood, those who drank surface water (HR = 0.94; 95% CI = 0.90–0.98) and tap water (HR = 0.80; 95% CI = 0.68–0.95) had a reduced risk of all-cause mortality in the total sample (**Table 3**). This indicates that, relative to drinking well water, the risks of death associated with drinking surface water and tap water were lower by 6% and 20%, respectively. The results were similar to the total population when stratified by sex, living area, and disease. When stratified by sex and living area, females drinking surface water had a lower risk of mortality (HR = 0.92; 95% CI = 0.87–0.97), as did the rural population drinking surface water compared to those used to drink well water (HR = 0.93; 95% CI = 0.87–0.99). Additionally, participants without any diseases who drank surface (HR = 0.91; 95% CI = 0.84–0.98) or tap water (HR = 0.75; 95% CI = 0.58–0.95) had a lower risk of mortality, although the association was significant only for spring water.

**Table 3:** Cox regression for all-cause mortality with water sources at childhood*

	Water sources at childhood, HR (95% CI)			
	**Well**	**Surface**	**Spring**	**Tap water**
**All participants**	ref	0.94 (0.90–0.98)	1.03 (0.95–1.13)	0.80 (0.68–0.95)
**Living area**				
Rural	ref	0.93 (0.87–0.99)	1.03 (0.91–1.18)	0.79 (0.62–1.01)
Urban	ref	0.95 (0.90–1.01)	1.03 (0.91–1.16)	0.83 (0.65–1.04)
**Sex**				
Male	ref	0.96 (0.90–1.03)	1.09 (0.94–1.28)	0.85 (0.71–1.01)
Female	ref	0.92 (0.87–0.97)	1.00 (0.90–1.12)	0.75 (0.47–1.19)
**Disease**				
No	ref	0.91 (0.84–0.98)	0.92 (0.78–1.10)	0.75 (0.58–0.95)
Yes	ref	0.96 (0.91–1.01)	1.08 (0.98–1.20)	0.85 (0.67–1.07)

CI – confidence interval, HR – hazard ratio, ref – reference

*Model adjusted for sex, age, residence, smoking, drinking alcohol, dietary diversity score, weight marital status, social activity and education, depression, MMSE, and disease.

### Association of kinds of water sources at around 60 years with the risk of mortality

Participants who drank tap water had a lower risk of mortality (HR = 0.81; 95% CI = 0.76–0.86), indicating a 19% reduction in the risk of death (Table S7 in the **Online Supplementary Document**). However, neither drinking surface water nor spring water was associated with mortality risk (*P* > 0.05). The results remained consistent across subgroups stratified by sex, living areas, and diseases.

### The association of kinds of water sources at present with the risk of mortality

Similar to the results for participants aged around 60 years, drinking tap water was associated with a 10% reduction in mortality risk (HR = 0.90; 95% CI = 0.86–0.94), whereas the consumption of spring and surface water no significant association (Table S8 in the **Online Supplementary Document**). In subgroup analyses, the association between drinking tap water and reduced mortality compared to well water was consistent with the overall results (*P* < 0.05). However, drinking spring water was marginally associated with higher mortality for participants living in urban areas (HR = 1.12; 95% CI = 1.01–1.24) or those with common diseases (HR = 1.11, 95% CI = 1.01–1.22).

### Sensitivity analysis

In sensitivity analyses, after imputing missing values, drinking unboiled water was associated with a higher mortality risk (HR = 1.10; 95% CI = 1.03–1.17). Drinking tap water was consistently associated with lower mortality risk across all three life stages (*P* < 0.05). Additionally, participants who drank surface water in childhood showed a reduced risk of mortality (HR = 0.93; 95% CI = 0.90–0.96). When individuals who died before the second wave (*i.e.* before 2005) were excluded, the results were comparable to those obtained using imputed models. However, participants who drank spring water at around 60 years (HR = 1.14; 95% CI = 1.01–1.29) or at present (HR = 1.27; 95% CI = 1.12–1.43) had a higher risk of mortality. Finally, after excluding participants with common diseases, drinking unboiled water remained a risk factor for mortality (HR = 1.11; 95% CI = 1.01–1.22). Drinking tap water was associated with reduced mortality at 60 years (HR = 0.80; 95% CI = 0.74–0.86) and at the time of data collection (HR = 0.88; 95% CI = 0.84–0.93), but not in childhood (Table S9 in the **Online Supplementary Document**). The time-varying analysis accounted for potential changes in drinking behaviors due to relocation or health status alterations, but found no significant association with all-cause mortality (Table S10 in the **Online Supplementary Document**). Furthermore, after adjusting for income level and geographical region – both established mortality risk factors – the association remained statistically significant (Table S11 in the **Online Supplementary Document**).

## DISCUSSION

To our knowledge, our study is the first to examine all-cause mortality associated with drinking habits and water sources among older people in China. The findings suggest that elderly individuals who habitually drank unboiled water had a higher risk of mortality. Compared to drinking well water, drinking tap water throughout all life stages significantly decreased mortality risk, as did drinking surface water in childhood. However, the association between drinking spring water and mortality varied by living area and disease status, with urban populations or individuals with common diseases facing higher risks.

Drinking preferences vary across countries and regions. In China, drinking boiled water is a common and culturally endorsed practice, with most participants reporting this habit [25]. Drinking unboiled or boiled water may mediate cognitive impairment and all-cause mortality among older adults in China [25,26]. For instance, the findings of one longitudinal study in China on 18 034 people aged >65 years suggested that long-term intake of unboiled water could increase the risk of cognitive impairment [24]. Another longitudinal study of 11 732 older adults linked accelerated cognitive decline to higher mortality, especially among persons aged 65–79 years and those with normal baseline cognition [26]. Unboiled water harbors bacteria, viruses, and parasites that can cause gastrointestinal diseases, which are particularly harmful to the elderly's vulnerable digestive systems [27]. Disturbances in gut microbiota are associated with conditions such as Alzheimer's disease, ischaemic stroke, diabetes, septicemia, inflammatory bowel disease, and osteoarthritis, all of which can elevate mortality risk in older populations [28–31]. A clinical trial in the USA involving 15 healthy subjects examined the effects of boiled water, chicken soup, and cold water on nasal mucus velocity and airflow resistance; its results showed that inhaling water vapor may temporarily increase nasal mucus velocity [32]. This suggests that drinking boiled water may potentially improve immunity, accelerate metabolism, and reduce the likelihood of upper respiratory tract infections compared to drinking unboiled water. However, a systematic review of six trials involving 387 participants found no conclusive evidence supporting the efficacy of heated water vapor (steam) for treating the common cold, citing high heterogeneity among the studies [33]. Further double-blind, randomised trials are needed to clarify these inconsistencies.

Several biological mechanisms explain how drinking unboiled water adversely affects older adults, with diarrhoea being the most immediate risk, as a USA study reported a 14.2% prevalence of acute diarrhoea among the elderly [34]. Diminished immune function makes older adults more susceptible to pathogens. *Vibrio cholerae*, for instance, induces severe diarrhoea by producing enterotoxins that elevate intracellular cyclic adenosine monophosphate, causing significant water and electrolyte loss [35,36]. Norovirus infects intestinal epithelial cells, triggering immune responses and inflammation, disrupting intestinal functions and leading to diarrhoea [37]. In amoebiasis, amoebic trophozoites destroy epithelial cells through contact lysis and phagocytosis, while secreted enterotoxins damage the intestinal mucosa. Moreover, they employ immune suppression and evasion mechanisms, further exacerbating intestinal inflammation [38]. However, *Vibrio cholerae*, norovirus and amoebae can all be killed by boiling [35,39,40]. A meta-analysis of 27 studies confirmed that boiled water significantly decreases the likelihood of *Vibrio cholerae*, Blastocystis, protozoal infections, viral infections, and nonspecific diarrhoeal diseases [41].

We found that drinking tap water in childhood, around the age of 60 years, and at the time of data collection reduces mortality risk among the elderly. Drinking tap water is one of the primary ways to consume plain water in modern China. Tap water typically has higher quality and cleanliness compared to well water, surface water, and spring water, owing to purification processes at treatment facilities. In 2001, the Chinese government issued the Standard for Drinking Water Hygiene, which set critical benchmarks for water quality in 2002 and beyond [42]. This standard outlines sanitary requirements for drinking water and its sources. In 2006, the Sanitary Standard for Drinking Water (GB 5749–2006) was introduced, encompassing 106 criteria, including microbial, toxicological, sensory, general chemical, and radioactive indicators [43]. These regulations have effectively controlled the spread of harmful microorganisms. Primary amoebic meningitis primarily results from exposure to untreated groundwater or untreated reservoir-sourced rainwater [44]. Conversely, exposure to tap water accounts for less than 2% of related incidents. The WHO posits that effective disinfection can be achieved under conditions where the pH is below 8.0, the temperature is 20°C, the contact time is at least 30 minutes, and the free chlorine residual concentration is 0.5 mg/L or higher [45]. However, tap water quality varies due to economic disparities. Economically developed regions, equipped with advanced water treatment infrastructure, use technologies such as membrane filtration and ozonation-activated carbon treatment [46,47], thereby ensuring higher tap water quality. To fully assess the long-term effects of tap water on all-cause mortality, future research should incorporate longer-term cohorts and more in-depth analysis [46].

Regional variations in contaminants such as heavy metals, pesticides, and microbial burdens in well water, surface water, and spring water significantly impact water quality and human health. A comprehensive research analysing river water quality across China from 2003 to 2018 revealed that eutrophication has alleviated and heavy metal pollution has gradually declined, with less than 0.3% of monitoring stations showing contamination [48]. Water pollution levels exhibited regional disparities, with 17.2% of the surveyed areas in eastern China affected, compared to 4.6% in the west, and the east coastal area being the most severely impacted at 24.4% [48]. Another cross-sectional study in rural China highlighted health risks from organic micropollutants and metals in groundwater, identifying 42 carcinogens (including 38 organic micropollutants) and finding cumulative carcinogenic risks exceeding acceptable limits (>10^−4^) at 34% of the sites, primarily attributed to metals, which contributed 79% (0–100%) of the risks [49].

We also found that drinking surface water during childhood was associated with a lower risk of all-cause mortality compared to drinking well water. In rural areas, excessive use of nitrogen fertilisers for crop cultivation often leads to nitrate levels in well water exceeding prescribed limits, with research linking fertiliser application to increased NO-3-N concentrations in water sources [50]. Elevated nitrate and nitrite levels in shallow well water have been linked to higher risks of gastric, colorectal, bladder, and kidney cancers [51–53]. Surface water, while possessing self-purification capacities to mitigate pollutants [54], has seen deteriorating quality in Chinese rivers over the past 20 years, challenging its purification capacity [55]. Long-term monitoring and further investigation into the dynamic changes in surface water quality are essential better to understand the association between drinking water and health outcomes.

However, we did not find an association between drinking spring water and all-cause mortality in the total sample in different life cycle periods. Interestingly, drinking spring water was linked to an increased mortality risk for urban residents and individuals with pre-existing diseases. It is worth noting that spring water, often a primary source for hilly and mountainous areas, can be polluted by excreta, livestock waste, and domestic wastewater [56–58].

This study has several limitations. Firstly, reliance on self-reported data introduces recall bias, as participants may inaccurately recall past drinking habits and details. Secondly, longitudinal design is susceptible to survivor bias, as healthier individuals are more likely to remain in the cohort over time. Thirdly, the small proportion of participants drinking spring water limited our statistical power; larger sample sizes should be included in future studies. Fourthly, the dataset we used lacked adequate and valid information on cause-specific mortality, which hindered us from exploring mechanisms linking drinking water to diseases such as chronic kidney disease, cancer, and respiratory diseases. Future collaborations between medical institutions and public datasets could provide more detailed disease-related data. Finally, the absence of detailed dietary data precluded analyses of associations between dietary patterns (*e.g.* daily water intake, beverage consumption) and mortality. Future studies should utilise objective water consumption data and historical water quality records to enhance accuracy.

## CONCLUSIONS

We found that elderly individuals drinking unboiled water face a higher risk of all-cause mortality. Drinking tap water is generally safer and healthier than well water throughout one’s life. Drinking surface water, when minimally polluted, is associated with lower mortality risk compared to well water. The relationship between drinking spring water and mortality requires further investigation. Our findings provide strong evidence to support public health initiatives, such as bolstering water treatment infrastructure and promoting the benefits of boiling water.

## Additional Material


Online Supplementary Document
