# Supplementary material for: The association between all-cause mortality with drinking habits and water sources: a nationwide longitudinal study on Chinese elderly
Source: J Glob Health. 2025 Sep 12;15:04120. doi: 10.7189/jogh.15.04120 (PMC12427601; doi:10.7189/jogh.15.04120)
Supplement: Online Supplementary Document [file jogh-15-04120-s001.pdf]

Supplement to: Shen S, Ma N, Wu T, Xiong Y, Yang J, Wu X, Xiang X. The association between all-cause mortality with drinking habits and water sources: a nationwide longitudinal study on Chinese elderly. J Glob Health. 2025;15:04120.

Table S1. Baseline characteristic with drinking habits

Characteristics	Boiled Water	Un-Boiled Water	*P*-Value
	N=14,952	N=711	
**Age(Years)**	86.5 (11.6)	86.1 (12.2)	0.47
**Weight**	49.1 (10.8)	46.3 (9.8)	<0.001
Sex			0.39
Male	6,386 (42.7%)	292 (41.1%)	
Female	8,566 (57.3%)	419 (58.9%)	
**Years of Schooling**			<0.001
Illiterate	9,179 (61.4%)	522 (73.4%)	
Primary School	4,233 (28.3%)	173 (24.3%)	
Higher School	1,540 (10.3%)	16 (2.3%)	
**Living Area**			<0.001
Rural	7,947 (53.2%)	548 (77.1%)	
City	7,005 (46.8%)	163 (22.9%)	
**Marital status**			
Never married	10,539 (70.49%)	536 (75.39%)	
Married	4,413 (29.51%)	175 (24.61%)	
**Drinking**			0.009
Never Drinking	9,994 (67.0%)	464 (65.4%)	
Formal Drinking	1,900 (12.7%)	72 (10.2%)	
Present Drinking	3,022 (20.3%)	173 (24.4%)	
**Smoking**			<0.001
Never Smoking	9,733 (65.2%)	478 (67.2%)	
Formal Smoking	2,463 (16.5%)	82 (11.5%)	
Present Smoking	2,728 (18.3%)	151 (21.2%)	
**Physical Activity**			<0.001
Low	8,438 (56.5%)	511 (72.0%)	

Medium	1,729 (11.6%)	58 (8.2%)	
High	4,756 (31.9%)	141 (19.9%)	
**Social Activity**			<0.001
Never	12,951 (86.6%)	664 (93.4%)	
Sometimes	1,726 (11.5%)	41 (5.8%)	
Always	275 (1.8%)	6 (0.8%)	
**Food Scores**			<0.001
Low	6,212 (41.5%)	194 (27.3%)	
Medium	4,244 (28.4%)	206 (29.0%)	
High	4,496 (30.1%)	311 (43.7%)	
**Cognitive Impairment**			0.054
No	5,413 (43.2%)	282 (47.2%)	
Yes	7,106 (56.8%)	315 (52.8%)	
**Depression**	11.8 (3.3)	12.5 (3.3)	<0.001
**Hypertension**			<0.001
No	12,037 (84.0%)	606 (89.0%)	
Yes	2,293 (16.0%)	75 (11.0%)	
**Diabetes**			<0.001
No	13,918 (97.5%)	679 (99.6%)	
Yes	357 (2.5%)	3 (0.4%)	
**Heart Attack**			<0.001
No	12,941 (90.5%)	650 (94.8%)	
Yes	1,352 (9.5%)	36 (5.2%)	
**Stroke/CVD**			0.31
No	13,555 (94.4%)	653 (95.3%)	
Yes	801 (5.6%)	32 (4.7%)	
**Pneumonia**			0.008
No	12,448 (86.4%)	616 (89.9%)	
Yes	1,966 (13.6%)	69 (10.1%)	
**Tuberculosis**			0.28
No	14,221 (99.2%)	682 (99.6%)	
Yes	116 (0.8%)	3 (0.4%)	

Table S2. Baseline characteristic by different water sources at childhood

Characteristics	Well	Surface	Spring	Tap Water	*P*-Value
	N=9,361	N=5,209	N=703	N=390	
**Age(Years)**	86.6 (11.6)	86.5 (11.6)	86.9 (11.9)	81.9 (11.4)	<0.001
**Weight**	49.3 (11.1)	48.4 (10.0)	45.8 (10.4)	54.5 (11.2)	<0.001
**Sex**					0.25
Male	4,022 (43.0%)	2,170 (41.7%)	309 (44.0%)	177 (45.4%)	
Female	5,339 (57.0%)	3,039 (58.3%)	394 (56.0%)	213 (54.6%)	
**Years of Schooling**					<0.001
Illiterate	5,802 (62.0%)	3,335 (64.0%)	461 (65.6%)	103 (26.4%)	
Primary School	2,653 (28.3%)	1,439 (27.6%)	186 (26.5%)	128 (32.8%)	
Higher School	906 (9.7%)	435 (8.4%)	56 (8.0%)	159 (40.8%)	
**Living Area**					<0.001
Rural	5,156 (55.1%)	2,841 (54.5%)	463 (65.9%)	35 (9.0%)	
City	4,205 (44.9%)	2,368 (45.5%)	240 (34.1%)	355 (91.0%)	
**Marital status**					<0.001
Never married	6,612 (70.63%)	3,717(71.36%)	526(74.82%)	220(56.41%)	
Married	2,749 (29.37%)	1,492(28.64%)	177(25.18%)	170(43.59%)	
**Drinking**					<0.001
Never Drinking	6,298 (67.4%)	3,415 (65.7%)	442 (62.9%)	303 (77.7%)	
Formal Drinking	1,185 (12.7%)	637 (12.3%)	114 (16.2%)	36 (9.2%)	
Present Drinking	1,855 (19.9%)	1,142 (22.0%)	147 (20.9%)	51 (13.1%)	
**Smoking**					0.24
Never Smoking	6,048 (64.7%)	3,438 (66.1%)	459 (65.3%)	266 (68.4%)	
Formal Smoking	1,528 (16.4%)	837 (16.1%)	113 (16.1%)	67 (17.2%)	
Present Smoking	1,769 (18.9%)	923 (17.8%)	131 (18.6%)	56 (14.4%)	
**Physical Activity**					<0.001
Low	5,038 (53.9%)	3,309 (63.6%)	484 (68.8%)	118 (30.3%)	
Medium	1,160 (12.4%)	509 (9.8%)	64 (9.1%)	54 (13.8%)	
High	3,142 (33.6%)	1,382 (26.6%)	155 (22.0%)	218 (55.9%)	
**Social Activity**					<0.001
Never	8,171 (87.3%)	4,523 (86.8%)	631 (89.8%)	290 (74.4%)	

Sometimes	1,016 (10.9%)	606 (11.6%)	58 (8.3%)	87 (22.3%)	
Always	174 (1.9%)	80 (1.5%)	14 (2.0%)	13 (3.3%)	
**Food Scores**					<0.001
Low	3,688 (39.4%)	2,197 (42.2%)	272 (38.7%)	249 (63.8%)	
Medium	2,617 (28.0%)	1,540 (29.6%)	208 (29.6%)	85 (21.8%)	
High	3,056 (32.6%)	1,472 (28.3%)	223 (31.7%)	56 (14.4%)	
**Cognitive Impairment**					<0.001
No	3,291 (42.3%)	2,069 (46.9%)	235 (40.2%)	100 (29.4%)	
Yes	4,490 (57.7%)	2,341 (53.1%)	350 (59.8%)	240 (70.6%)	
**Depression**	11.7 (3.3)	12.1 (3.2)	12.4 (3.1)	10.4 (3.3)	<0.001
**Hypertension**					<0.001
No	7,605 (84.4%)	4,167 (84.4%)	578 (85.8%)	293 (75.7%)	
Yes	1,405 (15.6%)	773 (15.6%)	96 (14.2%)	94 (24.3%)	
**Diabetes**					<0.001
No	8,776 (97.9%)	4,816 (97.6%)	656 (97.8%)	349 (90.2%)	
Yes	187 (2.1%)	120 (2.4%)	15 (2.2%)	38 (9.8%)	
**Heart Attack**					<0.001
No	8,104 (90.3%)	4,552 (92.0%)	623 (93.3%)	312 (80.6%)	
Yes	872 (9.7%)	396 (8.0%)	45 (6.7%)	75 (19.4%)	
**Stroke/CVD**					<0.001
No	8,475 (93.9%)	4,727 (95.4%)	648 (96.0%)	358 (92.5%)	
Yes	550 (6.1%)	227 (4.6%)	27 (4.0%)	29 (7.5%)	
**Pneumonia**					0.14
No	7,808 (86.2%)	4,315 (86.6%)	597 (88.6%)	344 (89.1%)	
Yes	1,248 (13.8%)	668 (13.4%)	77 (11.4%)	42 (10.9%)	
**Tuberculosis**					0.91
No	8,935 (99.2%)	4,913 (99.2%)	670 (99.4%)	385 (99.2%)	
Yes	70 (0.8%)	42 (0.8%)	4 (0.6%)	3 (0.8%)	

Table S3. Characteristic by different water sources at the age of 60 years

Characteristics	Well N=8,200	Surface N=2,480	Spring N=613	Tap Water N=4,370	*P*-Value
**Age(Years)**	87.2 (11.6)	89.1 (10.6)	87.9 (12.0)	83.2 (11.7)	<0.001
**Weight**	47.9 (10.3)	46.6 (9.6)	44.7 (9.1)	52.9 (11.5)	<0.001
**Sex**					<0.001
Male	3,397 (41.4%)	969 (39.1%)	258 (42.1%)	2,054 (47.0%)	
Female	4,803 (58.6%)	1,511 (60.9%)	355 (57.9%)	2,316 (53.0%)	
**Years of Schooling**					<0.001
Illiterate	5,560 (67.8%)	1,761 (71.0%)	432 (70.5%)	1,948 (44.6%)	
Primary School	2,138 (26.1%)	601 (24.2%)	147 (24.0%)	1,520 (34.8%)	
Higher School	502 (6.1%)	118 (4.8%)	34 (5.5%)	902 (20.6%)	
**Living Area**					<0.001
Rural	5,677 (69.2%)	1,510 (60.9%)	463 (75.5%)	845 (19.3%)	
City	2,523 (30.8%)	970 (39.1%)	150 (24.5%)	3,525 (80.7%)	
**Marital status**					<0.001
Never married	5,996 (73.12%)	1,939(78.19%)	468(76.35%)	2,672(61.14%)	
Married	2,204 (26.88%)	541(21.81%)	145(23.65%)	1,698(38.86%)	
**Drinking alcohol**					<0.001

Never Drinking	5,415 (66.2%)	1,584 (64.1%)	400 (65.4%)	3,059 (70.2%)	
Formal Drinking	1,005 (12.3%)	331 (13.4%)	95 (15.5%)	541 (12.4%)	
Present Drinking	1,760 (21.5%)	558 (22.6%)	117 (19.1%)	760 (17.4%)	
**Smoking**					<0.001
Never Smoking	5,339 (65.2%)	1,683 (68.0%)	405 (66.3%)	2,784 (63.8%)	
Formal Smoking	1,264 (15.4%)	390 (15.8%)	91 (14.9%)	800 (18.3%)	
Present Smoking	1,582 (19.3%)	402 (16.2%)	115 (18.8%)	780 (17.9%)	
**Physical Activity**					<0.001
Low	5,093 (62.2%)	1,689 (68.2%)	451 (73.8%)	1,716 (39.3%)	
Medium	858 (10.5%)	280 (11.3%)	43 (7.0%)	606 (13.9%)	
High	2,232 (27.3%)	506 (20.4%)	117 (19.1%)	2,042 (46.8%)	
**Social Activity**					<0.001
Never	7,367 (89.8%)	2,228 (89.8%)	553 (90.2%)	3,467 (79.3%)	
Sometimes	728 (8.9%)	230 (9.3%)	52 (8.5%)	757 (17.3%)	
Always	105 (1.3%)	22 (0.9%)	8 (1.3%)	146 (3.3%)	
**Food Scores**					<0.001
Low	2,867 (35.0%)	981 (39.6%)	217 (35.4%)	2,341 (53.6%)	
Medium	2,385 (29.1%)	771 (31.1%)	190 (31.0%)	1,104 (25.3%)	
High	2,948 (36.0%)	728 (29.4%)	206 (33.6%)	925 (21.2%)	

**Cognitive Impairment**					0.002
No	3,037 (44.4%)	920 (45.0%)	211 (42.1%)	1,527 (40.9%)	
Yes	3,804 (55.6%)	1,123 (55.0%)	290 (57.9%)	2,204 (59.1%)	
**Depression**	12.1 (3.3)	12.5 (3.1)	12.8 (3.0)	10.9 (3.3)	<0.001
**Hypertension**					<0.001
No	6,714 (86.2%)	2,021 (86.4%)	519 (88.3%)	3,389 (79.0%)	
Yes	1,079 (13.8%)	317 (13.6%)	69 (11.7%)	903 (21.0%)	
**Diabetes**					<0.001
No	7,645 (98.5%)	2,303 (98.5%)	579 (98.8%)	4,070 (95.3%)	
Yes	116 (1.5%)	35 (1.5%)	7 (1.2%)	202 (4.7%)	
**Heart Attack**					<0.001
No	7,266 (93.4%)	2,193 (93.6%)	557 (95.2%)	3,575 (83.7%)	
Yes	514 (6.6%)	149 (6.4%)	28 (4.8%)	697 (16.3%)	
**Stroke/CVD**					<0.001
No	7,415 (94.9%)	2,244 (95.7%)	577 (97.3%)	3,972 (92.5%)	
Yes	397 (5.1%)	100 (4.3%)	16 (2.7%)	320 (7.5%)	
**Pneumonia**					0.30
No	6,796 (86.5%)	2,048 (86.7%)	525 (89.0%)	3,695 (86.2%)	
Yes	1,062 (13.5%)	314 (13.3%)	65 (11.0%)	594 (13.8%)	
**Tuberculosis**					0.86

No	7,735 (99.2%)	2,328 (99.2%)	587 (99.5%)	4,253 (99.2%)
Yes	61 (0.8%)	19 (0.8%)	3 (0.5%)	36 (0.8%)

Table S4. Characteristic by different water sources at present

Characteristics	Well	Surface	Spring	Tap Water	*P*-Value
	N=8,200	N=2,480	N=613	N=4,370	
**Age(Years)**	87.2 (11.6)	89.1 (10.6)	87.9 (12.0)	83.2 (11.7)	<0.001
**Weight**	47.9 (10.3)	46.6 (9.6)	44.7 (9.1)	52.9 (11.5)	<0.001
**Sex**					<0.001
Male	3,397 (41.4%)	969 (39.1%)	258 (42.1%)	2,054 (47.0%)	
Female	4,803 (58.6%)	1,511 (60.9%)	355 (57.9%)	2,316 (53.0%)	
**Years of Schooling**					<0.001
Illiterate	5,560 (67.8%)	1,761 (71.0%)	432 (70.5%)	1,948 (44.6%)	
Primary School	2,138 (26.1%)	601 (24.2%)	147 (24.0%)	1,520 (34.8%)	
Higher School	502 (6.1%)	118 (4.8%)	34 (5.5%)	902 (20.6%)	
**Living Area**					<0.001
Rural	5,677 (69.2%)	1,510 (60.9%)	463 (75.5%)	845 (19.3%)	
City	2,523 (30.8%)	970 (39.1%)	150 (24.5%)	3,525 (80.7%)	
**Marital status**					

Never married	4,027(72.60%)	176(75.86%)	384(75.44%)	6,488(69.21%)	
Married	1,520(27.40%)	56(24.14%)	125(24.56%)	2,887(30.79%)	
**Drinking alcohol**					<0.001
Never Drinking	5,415 (66.2%)	1,584 (64.1%)	400 (65.4%)	3,059 (70.2%)	
Formal Drinking	1,005 (12.3%)	331 (13.4%)	95 (15.5%)	541 (12.4%)	
Present Drinking	1,760 (21.5%)	558 (22.6%)	117 (19.1%)	760 (17.4%)	
**Smoking**					<0.001
Never Smoking	5,339 (65.2%)	1,683 (68.0%)	405 (66.3%)	2,784 (63.8%)	
Formal Smoking	1,264 (15.4%)	390 (15.8%)	91 (14.9%)	800 (18.3%)	
Present Smoking	1,582 (19.3%)	402 (16.2%)	115 (18.8%)	780 (17.9%)	
**Physical Activity**					<0.001
Low	5,093 (62.2%)	1,689 (68.2%)	451 (73.8%)	1,716 (39.3%)	
Medium	858 (10.5%)	280 (11.3%)	43 (7.0%)	606 (13.9%)	
High	2,232 (27.3%)	506 (20.4%)	117 (19.1%)	2,042 (46.8%)	
**Social Activity**					<0.001
Never	7,367 (89.8%)	2,228 (89.8%)	553 (90.2%)	3,467 (79.3%)	

Sometimes	728 (8.9%)	230 (9.3%)	52 (8.5%)	757 (17.3%)	
Always	105 (1.3%)	22 (0.9%)	8 (1.3%)	146 (3.3%)	
**Food Scores**					<0.001
Low	2,867 (35.0%)	981 (39.6%)	217 (35.4%)	2,341 (53.6%)	
Medium	2,385 (29.1%)	771 (31.1%)	190 (31.0%)	1,104 (25.3%)	
High	2,948 (36.0%)	728 (29.4%)	206 (33.6%)	925 (21.2%)	
**Cognitive Impairment**					0.002
No	3,037 (44.4%)	920 (45.0%)	211 (42.1%)	1,527 (40.9%)	
Yes	3,804 (55.6%)	1,123 (55.0%)	290 (57.9%)	2,204 (59.1%)	
**Depression**	12.1 (3.3)	12.5 (3.1)	12.8 (3.0)	10.9 (3.3)	<0.001
**Hypertension**					<0.001
No	6,714 (86.2%)	2,021 (86.4%)	519 (88.3%)	3,389 (79.0%)	
Yes	1,079 (13.8%)	317 (13.6%)	69 (11.7%)	903 (21.0%)	
**Diabetes**					<0.001
No	7,645 (98.5%)	2,303 (98.5%)	579 (98.8%)	4,070 (95.3%)	
Yes	116 (1.5%)	35 (1.5%)	7 (1.2%)	202 (4.7%)	
**Heart Attack**					<0.001

No	7,266 (93.4%)	2,193 (93.6%)	557 (95.2%)	3,575 (83.7%)	
Yes	514 (6.6%)	149 (6.4%)	28 (4.8%)	697 (16.3%)	
**Stroke/CVD**					<0.001
No	7,415 (94.9%)	2,244 (95.7%)	577 (97.3%)	3,972 (92.5%)	
Yes	397 (5.1%)	100 (4.3%)	16 (2.7%)	320 (7.5%)	
**Pneumonia**					0.30
No	6,796 (86.5%)	2,048 (86.7%)	525 (89.0%)	3,695 (86.2%)	
Yes	1,062 (13.5%)	314 (13.3%)	65 (11.0%)	594 (13.8%)	
**Tuberculosis**					0.86
No	7,735 (99.2%)	2,328 (99.2%)	587 (99.5%)	4,253 (99.2%)	
Yes	61 (0.8%)	19 (0.8%)	3 (0.5%)	36 (0.8%)	

Table S5. Region characteristic with drinking habits

**Region**	**Drinking habits**	
	**Boiled water**	**Un-boiled water**
**Northern China**	2,862	215
**Eastern China**	6,395	111
**Southern China**	4,006	346
**Western China**	424	8
**Missing**	1,265	31
Total	14,952	711

Table S6. Region characteristic by different water sources at the age of 60 years

Region	Water sources at 60 years			
	Well	Surface	Spring	Tap water
**Northern China**	1,767	181	30	1,099
**Eastern China**	2,966	1,293	217	2,030
**Southern China**	2,278	912	315	847
**Western China**	243	13	24	152
**Missing**	946	81	27	242
**Total**	8,200	2,480	613	4370

Table S7. Hazard ratio (95%CI) for all-cause mortality with water sources at 60 years

All-cause Mortality	Water Sources at 60 Years			
	**Well**	**Surface**	**Spring**	**Tap Water**
**All Participants**	1.00	0.99(0.94,1.04)	1.01(0.92,1.10)	**0.81(0.76,0.86)**
**Living Area**				
Rural	1.00	1.01(0.93,1.11)	0.94(0.77,1.14)	**0.81(0.75,0.87)**
City	1.00	0.97(0.91,1.04)	1.04(0.93,1.16)	**0.85(0.77,0.94)**
**Sex**				
Male	1.00	0.96(0.88,1.04)	0.96(0.84,1.10)	**0.76(0.69,0.82)**
Female	1.00	1.01(0.95,1.09)	1.05(0.93,1.18)	**0.86(0.80,0.94)**
**Disease**				
No	1.00	0.95(0.86,1.05)	0.97(0.81,1.15)	**0.84(0.76,0.92)**
Yes	1.00	1.01(0.94,1.07)	1.02(0.92,1.14)	**0.80(0.74,0.86)**

Model adjusted for sex. age, residence, smoking, drinking alcohol, dietary diversity score, BMI, weight, marital status, social activity and education, depression, MMSE, and disease.

Table S8. Hazard ratio (95%CI) for all-cause mortality with water sources at present

All-cause mortality	water sources at present			
	Well	Surface	Spring	Tap water
All participants	1.00	0.98(0.84,1.14)	1.08(0.99,1.18)	**0.90(0.86,0.94)**
Living area				
Rural	1.00	0.96(0.72,1.29)	1.01(0.81,1.27)	**0.84(0.78,0.91)**
City	1.00	0.98(0.81,1.18)	**1.12(1.01,1.24)**	**0.92(0.87,0.97)**
Sex				
Male	1.00	0.95(0.75,1.20)	1.10(0.96,1.25)	**0.82(0.77,0.88)**
Female	1.00	0.98(0.81,1.20)	1.06(0.94,1.20)	**0.84(0.79,0.89)**
Disease				
No	1.00	0.99(0.76,1.28)	1.01(0.83,1.22)	**0.85(0.79,0.92)**
Yes	1.00	0.97(0.81,1.17)	**1.11(1.01,1.22)**	**0.82(0.78,0.87)**

Model adjusted for sex. age, residence, smoking, drinking alcohol, dietary diversity score, BMI, weight, marital status, social activity and education, depression, MMSE, and disease.

Table S9. Sensitivity analyses for the association of drinking habits and water sources with 8-year mortality

**Characteristics**	**Hazard ratio (95%)**		
**Drinking habit**	Model 1 *	Model 2 †	Model 3‡
Boiled water	Ref.	Ref.	Ref.
Un -boiled water	**1.099(1.03,1.17)**	**1.31(1.18,1.45)**	**1.11(1.01,1.22)**
**Water sources at childhood**			
Well	Ref.	Ref.	Ref.
Surface	**0.93(0.90,0.96)**	**0.92(0.86,0.98)**	0.96(0.91,1.01)
Spring	0.99(0.92,1.06)	1.13(1.01,1.28)	1.08(0.98,1.20)
Tap water	**0.85(0.74,0.98)**	**0.69(0.54,0.89)**	0.85(0.67,1.07)
**Water sources at 60 years**			
Well	Ref.	Ref.	Ref.
Surface	0.97(0.93,1.01)	0.99(0.91,1.07)	1.01(0.94,1.07)
Spring	0.97(0.90,1.04)	**1.14(1.01,1.29)**	1.00(0.92,1.14)
Tap water	**0.81(0.77,0.85)**	**0.71(0.66,0.77)**	**0.80(0.74,0.86)**
**Water sources at present**			
Well	Ref.	Ref.	Ref.
Surface	0.96(0.86,1.08)	1.14(0.93,1.39)	0.97(0.81,1.18)
Spring	1.04(0.97,1.11)	**1.27(1.12,1.43)**	1.12(1.01,1.23)
Tap water	**0.89(0.86,0.93)**	**0.81(0.76,0.87)**	**0.88(0.84,0.93)**

In models 1, 2, and 3. we adjusted for sex. age, residence, smoking, alcohol drinking, dietary diversity score, weight, marital status, social activity and education, depression, MMSE and chronic disease

* Multiple imputations conducted for missing data

† Exclusion of deaths that occurred in the first year of follow-up.

‡ Exclusion of participants with a history of the disease

Table S10. Time-varying analyses for all-cause mortality with drinking habits.

All-cause mortality	Drinking habits	
	Boiled water	Un-boiled water
**Number of deaths**	10079	559
**Person-years**	133590	5921
**Incident rate (per 1000)**	75.45	94.41
**All participants**	1.00	1.00(0.92,1.09)
**Living area**		
Rural	1.00	0.99(0.83,1.18)
City	1.00	1.01(0.91,1.11)
**Sex**		
Male	1.00	1.11(0.98,1.26)
Female	1.00	0.93(0.83,1.05)
**Disease**		
No	1.00	0.97(0.88,1.07)
Yes	1.00	1.10(0.94,1.30)

Model adjusted for baseline sex. age, residence, smoking, drinking alcohol, dietary diversity score, weight, marital status, social activity and education, depression, MMSE, and disease.

Table S11. Hazard ratio (95% CI) for all-cause mortality with drinking habits further adjusted for income, region, and disease.

All-cause mortality	Drinking habits	
	Boiled water	Un-boiled water
**Number of deaths**	10079	559
**Person-years**	133590	5921
**Incident rate (per 1000)**	75.45	94.41
**All participants**	1.00	**1.16(1.08,1.26)**
**Living area**		
Rural	1.00	**1.23(1.04,1.46)**
City	1.00	**1.16(1.06,1.27)**
**Sex**		
Male	1.00	**1.19(1.05,1.34)**
Female	1.00	**1.15(1.04,1.27)**
**Disease**		
No	1.00	**1.12(1.02,1.24)**
Yes	1.00	**1.28(1.11,1.49)**

Model adjusted for baseline sex. age, residence,income,region, smoking, drinking alcohol, dietary diversity score, weight, marital status, social activity and education, depression, MMSE, and disease
